# Local Spatialized Knowledge of Threats to Forest Conservation in Ghana’s High Forest Zone

**DOI:** 10.1007/s00267-021-01455-0

**Published:** 2021-03-22

**Authors:** Dorcas Peggy Somuah, Mirjam A. F. Ros–Tonen, Isa Baud

**Affiliations:** 1 Department of Forest Resources Technology, Kwame Nkrumah University of Science and Technology (KNUST), Kumasi, Ghana; 2 Department of Geography, Planning and International Development Studies, University of Amsterdam, Amsterdam, The Netherlands

**Keywords:** Participatory mapping, Participatory geographic information systems, Spatialized community knowledge, Inclusiveness, High forest zone, Ghana

## Abstract

Although deforestation rates are declining, protected forest areas remain under threat. While the importance of spatialized (‘mapped’) community knowledge for conservation planning is acknowledged in scientific literature, the integration of such knowledge in forest governance and conservation planning remains scarce, particularly in Ghana. This paper aims to make clear how participatory spatial knowledge tools and geographic information systems can be used to assess the threats to forest conservation in Ghana’s high forest zone. The results show that holders of spatial community-embedded knowledge not only sketch-mapped the location and spatial distribution of the threats to forest conservation in the forest reserves, but also provided information on the actors they perceived to be causing such threats. Such information is not available in forest inventories conducted by the responsible government agencies, but is needed to focus conservation strategies and make them more effective. Maps with the anticipated condition of the forest in 10 years’ time furthermore provided insights which can help governance actors to deal with the underlying drivers of forest degradation. This suggests that local spatialized knowledge needs to be integrated into the institutional arrangements for the governance of forested landscapes, and that such governance cannot be effective without the inclusion of local people’s knowledge. Due consideration is however to be given to the conditions that ensure that spatialized knowledge production and its use in landscape management decision-making occurs in an inclusive manner.

## Introduction

Despite declining deforestation rates, the world’s forests reduced by an average rate of 10 million hectares between 2015 and 2020, raising concerns about biodiversity loss, freshwater supply, negative health effects, climate change and the livelihoods of forest-dependent people (FAO 2020a, 2020b:13). The need to engage actors from multiple sectors and levels in the governance and management of forested landscapes is widely acknowledged (Mwangi and Wardell 2013; Arts et al. 2017; Ros-Tonen et al. 2018). Especially the need to “mobilize, translate, negotiate, synthesize and apply” indigenous and traditional knowledge (Tengö et al. 2017:13) is considered vital for environmental governance (Díaz et al. 2015; Brondizio and Le Tourneau 2016). This is also acknowledged in the United Nations Convention on Biological Diversity (CBD) and recent reports of the Intergovernmental Science-Policy Platform on Biodiversity and Ecosystem Services (IPBES 2019) and the Global Environmental Outlook of the United Nations Environmental Program (UNEP 2019). However, in practice, the views and knowledge of indigenous and local communities are hardly taken seriously in forest and environmental governance and efforts to do so have been qualified as “lip service” (Brondizio and Le Tourneau 2016) or “tokenism” (Somuah 2018). This applies even more to local spatialized knowledge—defined here as a combination of context-embedded community knowledge and codified knowledge based on the use of Geographic Information Systems (GIS) to develop maps in order to understand spatial processes such as deforestation and drivers of deforestation; often done through a participatory approach (van Ewijk and Baud 2009; Somuah 2018). Ignoring or excluding local spatialized knowledge in conservation and land-use planning occurs despite its importance for localizing threats to protected areas and identifying areas that are key to local people’s livelihoods and use of environmental services (Angelstam et al. 2013; Ramirez-Gomez et al. 2016; Delgado-Aguilar et al. 2019; Ioki et al. 2019).

Ghana is one of the countries where recognition of local spatialized knowledge is completely absent in the implementation of forest policies and conservation planning (Somuah 2018). However, several studies have applied participatory geographical information systems (PGIS) or participatory mapping processes in the Ghanaian forest context—as education tools for conflict resolution in collaborative forest management (CFM) programs (Kyem 2004, 2006); community empowerment for protecting local forest resources (Kyem 2001); and assessment of environmental degradation (Agyemang et al. 2007). This study and those by (Asubonteng et al. 2021, this issue) and Aggrey et al. (under revision for this issue) are the first studies on Ghana that explicitly indicate how spatialized knowledge can contribute to inclusive conservation planning and landscape governance. Spatializing knowledge involves linking different types of community knowledge to geographic locations on maps and analyzing their spatial relationships. This can be done by using geospatial technologies such as GIS (Baud et al. 2011; Pfeffer et al. 2013). Spatializing knowledge involves two phases: developing the methodologies for producing spatial information and knowledge, and then analyzing the role that mapping can play in generating a better understanding of locationally bound situations and transparent decision-making in natural resource governance (c.f. Pfeffer et al. 2013). Generally, the methodologies through which knowledge is produced, used, and exchanged include: (i) the knowledge-generation process and the degree of participation or contestation involved, (ii) the adoption of geospatial tools (GIS) that enable spatial data collection, processing, analysis and visualization, and (iii) the societal purpose of knowledge generation, which might be the inclusion of marginalized groups to contribute to inclusive landscape governance (Baud et al. 2011; Opdam et al. 2013; Pfeffer et al. 2013; Nel et al. 2016; Fagerholm et al. 2019; Asubonteng et al. 2021, this issue; Aggrey et al. under revision, this issue).

The main question addressed in this article is: how can participatory spatial knowledge tools and PGIS improve (or contribute to) an integrated assessment of threats to forest conservation in Ghana’s high forest zone and what conditions need to be met to ensure the inclusiveness of spatialized knowledge production and its use for forest governance? To answer this question, the next section first provides background to Ghana’s conservation context. Then we explain the way in which participatory spatial knowledge production tools were applied. The results section shows how local spatialized knowledge is laid down in maps using participatory mapping and geospatial tools, and what the maps reveal about current and anticipated threats to protected areas. The discussion focuses on the question of what contributions the application of participatory spatial knowledge tools and the use of spatialized community-embedded knowledge make toward more integrated forest landscape governance.

## Ghana’s Conservation Context

Policies and institutional arrangements for the governance of Ghana’s forests have evolved since colonial forestry (Amanor 2004; Derkyi 2012). Forest and wildlife reserves were created during the pre-independence era (1908–1948), mainly to serve the timber interests of the British colonizers (Oduro et al. 2011). Subsequently these reserves were governed hierarchically with little or no consideration for the rights of forest-dependent people (Derkyi 2012). Forestry institutions assumed that “local people have no worthwhile knowledge and interest in the conservation or protection of forests” (Kotey et al. 1998: 12). Thus, the only form of forestry that was considered meaningful was the one technocratically practiced by the forester, focusing on timber production and protecting forests against “destructive agencies” (read: people) (Ibid., p.11).

The post-independence 1994 Forest Policy sought to address the needs of forest-dependent people, which the previous policies failed to do. Its legislative instruments enshrine community participation as a central tenet of forestry policy implemented through a CFM approach, marking a shift from authoritarian control to more inclusive processes through stakeholder involvement (Sasu 2005; Brown and Amanor 2006). A new Forest and Wildlife Policy was launched in 2012 to set new policy targets to combat ongoing deforestation and to guide the implementation of and coordination with multilevel forest governance initiatives to which Ghana had committed (Adom 2017). These international commitments include the Voluntary Partnership Agreement with the European Union/Forest Law Enforcement Governance and Trade (VPA/FLEGT) to combat illegal logging; the National Forest Program partnership between Ghana and the Food and Agricultural Organization of the United Nations (FAO), and the implementation of REDD + policies (Reducing Emissions from Deforestation and forest Degradation, including the role of conservation, sustainable forest management and enhancement of forest carbon stocks (Derkyi 2012; Adom 2017). The policy explicitly mentions “Integrating traditional and scientific knowledge to promote sustainable forest management” and “the involvement of forest-fringe communities” among its guiding principles (p. 10–11), and includes a strategic direction to “Promote the traditional autonomy for the protection and management of sacred forests and cultural heritage sites” (p.18). However, rather than valuing local knowledge, the focus of knowledge exchange with communities is on training, capacity building and dissemination of information, and “document[ing] sacred natural sites of biological, spiritual, religious, cultural and heritage values whilst maintaining their secrecy where required” (p.18).

Despite well-intended policies and international cooperation to improve forest governance, current forest resources continue to suffer from ongoing deforestation and forest degradation (Box 1). Many forest and wildlife reserves as well as scared groves are heavily encroached and degraded (Acheampong et al. 2019; Adom et al. 2019; Ayivor et al. 2020). This also applies to the so-called Globally Significant Biodiversity Areas (GSBAs), demarcated within forest reserves by the Ministry of Land and Natural Resources (MLNR) in the early 2000s for permanent protection (Derkyi et al. 2013). These threats have been attributed to illegal logging and chainsaw milling, the expansion of cocoa and oil palm, and the non-operationalization of formulated participatory management plans (Decher and Fahr 2005; Kyereh et al. 2006; Derkyi 2012; Derkyi et al. 2013; Asubonteng et al. 2018; Acheampong et al. 2019).

Local spatialized knowledge is relevant for policies that aim to address such pressures on protected areas, because it provides knowledge concerning the spatially concentrated use and management of natural resources in areas that may or may not yet have been identified by formal forestry as vulnerable; provides knowledge that can be combined with other knowledge for more integrated and informed decision-making; and recognizes existing local knowledge and contributes to active participation and empowerment of local people (Charnley et al. 2008; Raymond et al. 2010; Carvalho and Frazão-Moreira 2011; Padmanaba et al. 2013; Corbett et al. 2016).

Box 1 Ghana’s contested deforestation figures Controversy exists about Ghana’s recent deforestation rates since Forest Watch and the World Resources Institute reported that Ghana lost 8% of its humid primary forest between 2000 and 2019 (Global Forest Watch 2019) and ranked Ghana first among the top 10 countries losing primary forest after an increase in primary forest loss of 60% from 2017 to 2018 (Weisse and Dow Goldman 2019). In contrast, the FAO mentions an annual deforestation rate of 0.05% per year (4300 ha/year) for 2010–2020 (FAO 2020b) and the Ministry of Lands and Natural Resources (MLNR 2016) even claims an increase in forest cover of 0.3% per year thanks to its reforestation program, reduction of forest fires, and natural regeneration. Notwithstanding these “conflicting truths” (Kansanga et al. 2019), Ghana’s forest reserves and protected areas are heavily degraded (Addo-Fordjour and Ankomah 2017; Acheampong et al. 2019; Adom et al. 2019) and little closed forest remains outside Ghana’s forest reserve network (MLNR 2016).

## Methods and Materials

This section first explains the rationale behind the selection of the study sites and participants. Next, we make the criteria for the inclusion or exclusion of participants in the knowledge-generation process explicit. The third sub-section elaborates on the participatory mapping and geospatial tools used to produce maps for further analysis. Finally, we elaborate on the ethical considerations involved.

### Study Areas

Two forest reserves were selected for a comparative analysis based on the following criteria: (i) degree of protection status—one totally protected forest reserve (Apedwa) and one partially protected forest reserve (Tano-Offin); (ii) coverage of the main management regimes in the partially protected reserve, i.e., conservation, production, and reforestation^1^; (iii) degree of forest degradation with one relatively well-preserved forest reserve (Tano-Offin) and one degraded forest reserve in order to compare challenges to conservation; and (iv) location in Akan-speaking regions because the first author, who collected the data, speaks the local language (Twi), which facilitated communication and trust.

**Fig. 1:**
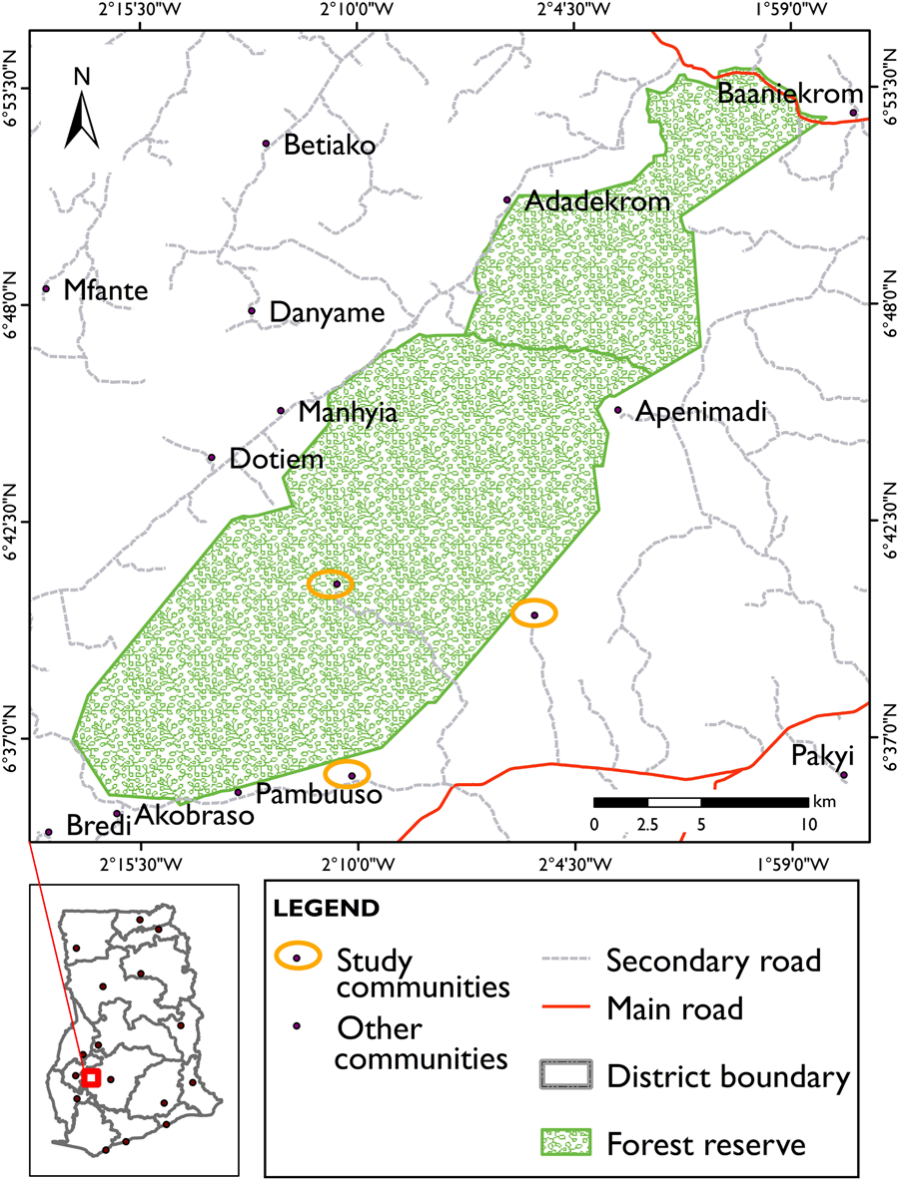
Map of the Tano-Offin forest reserve (partially protected), showing the study communities (*Source:* Somuah 2018). The inset map indicates the location of the study area within Ghana

The partially protected Tano-Offin reserve (Fig. 1) was selected because it has both a GSBA and a production and reforestation area. Moreover, studies carried out in and around the reserve (Derkyi 2012; Derkyi et al. 2013) indicate degradation due to illegal logging, chainsaw milling, farming, and non-operationalization of participatory management plans. Nonetheless, this reserve is relatively well preserved compared to other forest reserves (pers. comm. Kyereh et al. 2006). An additional advantage of selecting this forest reserve is that it contains an admitted village (Kyekyewere). This gives it unique characteristics compared to other forest reserves under partial coverage protection and also provides the opportunity to map local spatialized knowledge on forest use, conservation, and its challenges under different management regimes within the same forest reserve. The Tano-Offin forest reserve is located in the Ashanti Region of Ghana between latitudes 60^°^54’ and 60^°^35’ North and longitudes 10^°^57’ and 20^°^17’ West (Kyereh et al. 2006). It covers a total area of 413.92 km^2^ out of which 178.34 km^2^ (44.5%) constitutes the GSBA. It is one of the three upland evergreen forests; a rare forest type in Ghana and exceptional in terms of floral richness and diversity.

**Fig. 2:**
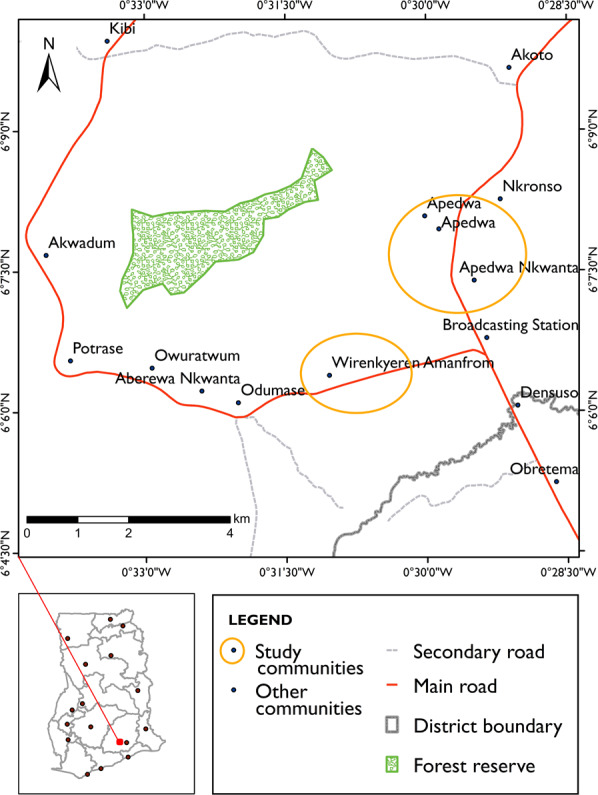
Map of the Apedwa forest reserve (total coverage protection) showing the study communities. The inset map indicates the study area location within Ghana. (*Source*: Somuah 2018)

The Apedwa reserve (Fig. 2) was selected as the reserve under total coverage protection, enabling a comparative analysis of the threats to forest conservation between forest reserves of different conservation status. The forest reserve lies between latitudes 6^°^06’ and 6^°^35’ North and longitudes 0^°^16’ and 0^°^42’ W and covers an area of 410 ha with a total perimeter of 12.65 km. The entire reserve was declared a GSBA in 1999, so there are no timber harvesting rights. The Apedwa forest reserve was classified as an upland evergreen forest because of its existence on isolated hills (between 500 and 750 m elevation), located within the moist semi-deciduous forest type. Trees that occur in this vegetation type reach a maximum height of 45 meters.

Criteria for the selection of specific study villages included accessibility, willingness of village authorities to participate in the research, and active involvement in forest conservation, as evidenced by the presence of actively functioning collaborative management groups such as the community biodiversity advisory groups (CBAGs) and the community fire volunteer squads (CFVS).^2^ In the Tano-Offin forest we selected reserve three communities from the different management regimes, namely Akantanso and Kyereyaaso from the production regime and Kyekyewere from a strictly protected regime (the GSBA). Kyereyaaso and Akantanso were selected based on findings from a reconnaissance survey of the area with assistance of a range supervisor and a forest guard^3^ from the Forest Services Division (FSD). Kyekyewere was selected due to its legal status as an ‘admitted village’ that was allowed at the time of demarcation of the GSBA in the early 1990s.

In the totally protected forest reserve (Apedwa) we selected two communities (Amanfrom and Apedwa) based on their location within the reserve, accessibility, willingness of village authorities to participate in the research and active community involvement in forest conservation. Reconnaissance studies in the study areas also revealed degradation due to illegal chainsaw activities, which had led to canopy gaps and a significant decrease in forest cover. Table 1 shows further details in terms of adult population in the study communities.

**Table 1:** Estimated adult population in the Tano-Offin and Apedwa reserves

Forest reserve	Management regime	Study community	Adult population		
			Male	Female	Total
Tano-Offin	Production	Akantanso	438	406	844
	Strict protection	Kyekyewere	485	386	871
	Production	Kyereyaaso	472	504	976
Apedwa	Strict protection	Amamfrom	348	852	1400
	Strict protection	Apedwa	3301	5200	8501

*Source:* Ghana Statistical Service (2010)

### Selection of Local Spatial Knowledge Holders

The research focused specifically on holders of local spatial knowledge, i.e., knowledge that is place-based and acquired by communities/citizens in their close contact with a specific area or resource (McCall 2021) and as they move around and observe the surrounding space in their environment (Ishikawa and Montello 2006; van Ewijk and Baud 2009; Somuah 2018; McCall 2021). When mapped (“spatialized”), this type of knowledge can become partly codified and exchanged for particular purposes (van Ewijk and Baud 2009: 220). In each village, the chief and elderly men and women selected six knowledge holders without interference from the researcher. However, the researcher provided criteria for defining local spatial knowledge holders as those community members with membership of the CBAGs, Community Fire Volunteers (CFVs) or having rich spatial knowledge of their environment. Based on this, community consultations began in February 2014 with community gatherings in the selected villages, involving the chiefs, elders, CBAGs, CFVs, range supervisors, and community members. These meetings were meant to formally introduce the research and to seek their consent to take part in the research. This was followed by community meetings in the study areas in July 2014 to identify relevant occupational groups present in the communities and identify and list members of each of them. In addition to the groups identified above, these included Unit Committees^4^, hunters, traders, farmers, chief and elders, artisanal millers, and chainsaw operators. Each community member was identified by his/her primary occupation in order to avoid duplication. After identification, members belonging to the occupational groups considered relevant as a source of local spatialized knowledge were selected purposively with the assistance of community leaders. Consideration was given to members currently resident in the community who were readily available for the interviews. Surveys were later conducted to validate findings from the p-mapping and PGIS exercise. Purposive sampling was used to select community members for the survey interviews.

### Participatory Mapping and Other Geospatial Tools Used

The participatory spatial knowledge tools adopted for this study were sketch mapping and transect mapping.^5^ These were combined with Global Positioning System (GPS) mapping and scale mapping.^6^ The rationale for combining the various tools was to visualize the types of knowledge in a geospatial environment (GIS) for broader exchange and use. The participatory tools enabled the knowledge of the various actors identified in the study areas to be included in the mapping process. Prior to data collection, the knowledge holders were trained in mapping techniques, basic image interpretation, ethics of PGIS and the use of the GPS (Garmin). The training was done to ensure that comparable information was mapped from the sites in the study area and for mappers to develop team cohesion and trust. The training also provided the opportunity to determine whether the p-mapping tools were feasible and to utilize the knowledge of the community knowledge holders to refine the tools. After the training, each group of knowledge holders produced maps of their various communities, indicating the location of contemporary (2014) and anticipated (2024) threats to forest conservation and forest-cover change.^7^ Colored pens, pencils, and erasers were used as material. The maps generated through local spatialized knowledge production indicate locations of forest resources, including medicinal plants and tree species of economic value. However, the participants did not disclose plant species and special sites of biocultural importance such as sacred groves during the mapping process, in order to prevent outsiders interfering with them. Moreover, the forest resources indicated on the maps are not accessible without consulting people from the study communities. For example, special rites need to be performed before one can access *Okoubaka aubrevillei* and *Spiropetalum heterophyllum* for medicinal purposes, as they are considered sacred. As such, the maps are of limited use for direct benefits for others than community members.

Transect walks were taken through the forest based on the information provided in the sketch maps to verify the features on the maps and measure coordinates of the relevant observation points with the GPS. Validation meetings were held with community members to verify the information provided on the maps.

A survey was conducted among 598 inhabitants in the study communities to validate the findings from the p-mapping exercise and to provide information on the socio-economic characteristics of the study communities. In addition, the survey provided information on groups of people causing threats to forest conservation and their origin to serve as quantitative added value to the visualized location of threats.

After the p-mapping exercise, the sketch maps were scanned to link the knowledge produced in a particular forest reserve in a geospatial environment. The digitized features were made to mimic ground features, using appropriate symbols to relate maps to ground features. The final maps, which indicated the locations of threats to forest conservation, were prepared and exported to picture format for further analysis and interpretation.

### Ethical Concerns

Issues of ethical concern for the knowledge generation process included how participants were recruited for this research, whether their rights were respected, and whether care was taken in maintaining confidentiality of records.

Free, Prior and Informed Consent (FPIC) is a central tenet in research involving local people, indigenous communities, and local knowledge. FPIC requires that consent be sought from the participants before the research begins. In this study, consent was sought verbally from the communities regarding how, when, and where results of the research will be published. At community meetings the purpose of the research was explained and permission sought to conduct the research. Consent was also sought to use audio-visual material during data collection and to publish maps generated from p-mapping and PGIS. Community leaders were asked to select spatial knowledge holders. No efforts were made to influence the decision of the communities to participate in the research or the selection of the spatial knowledge holders in the community. A timeframe of 14 days was given after which the community leaders would inform the FSD forest guard of their decision. The forest guard then informed the first author of the outcome. Areas that communities did not permit to be included in the research, such as sacred natural sites, were not documented. In addition, the names of places which community members did not want to disclose were excluded from the p-mapping exercise and coordinates were not provided to that effect. The community members agreed that the findings of the research would be published in English, which is Ghana’s official language.

During the training of local spatial knowledge holders, the data collection methods such as questionnaires, observations, and use of audio-visual material were explained. Verbal consent of the respondents was sought before the survey questionnaires were administered and used. After consent, respondents were free to withdraw if they decided not to participate any longer. However, after having given consent none of the respondents withdrew from the data collection process.

A protocol was observed in accordance with the customary traditions, which required that one presents drinks (Dutch gin) on the first visit to the chief’s palace. Spatial knowledge holders and respondents to the survey were compensated for their time spent. For spatial knowledge holders, this was calculated as the money gained per day by working on their farmlands. Agreement on the amount paid was reached through discussions with the knowledge holders and sub-chiefs. Each respondent to the survey was handed a bar of soap as a compensation for time input—a practice which is common in Ghana and appreciated by the community members.

Finally, participants were assured of confidentiality of identity. This was done by ensuring that the names of the respondents would be withheld from publications resulting from the research. We therefore refer to names of communities instead of those of individuals. No permission was granted to outsiders to deal with the raw data in order to protect the respondents. Maps showing the location of illegal farms within the forest reserves were validated in the presence of a forest guard from the FSD. These validation meetings made clear that the forest guard was well aware of these locations as the so-called rapid patrol team (made up of the military and FSD officials) had already destroyed such farms in previous operations; hence such sites did not provide new information to the FSD to act on. Foreseeable beneficiaries were explained as the international academic community, the spatial knowledge holders from the selected communities, and state and non-state organizations at the local, national, and global levels.

## Results

This section first presents how the local spatial knowledge holders from both forest reserves visualize the main threats to forest conservation in their communities with the aid of the maps generated in the knowledge production process. Further, a comparative analysis of the two forest reserves and different management regimes, brings out minor threats and their underlying causes. The last section shows how mapping the anticipated future condition of the forest reserves presents an added management value to forest conservation and integrated landscape governance.

### Spatializing the Major Threat to Forest Conservation: Chainsaw Milling

**Fig. 3:**
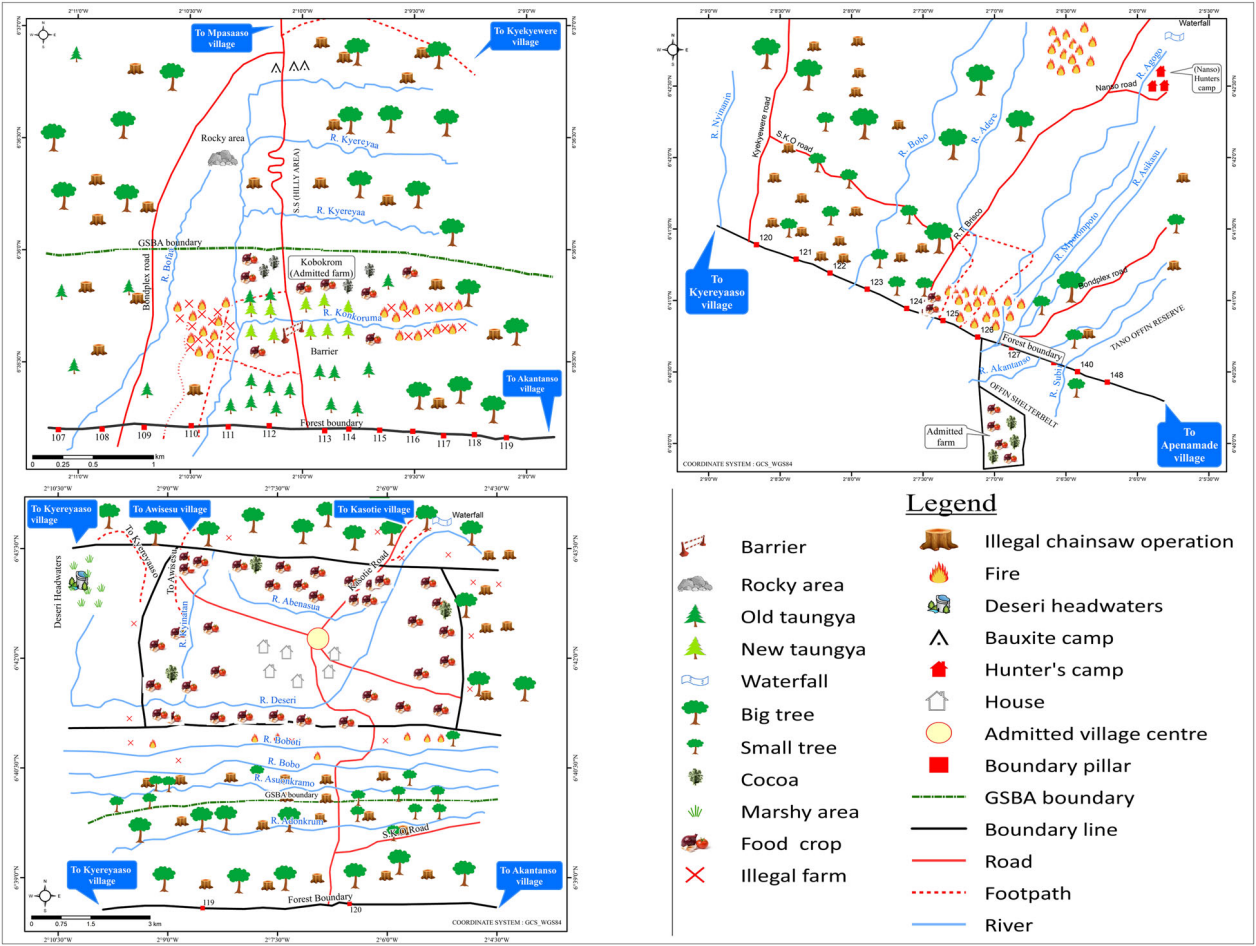
Contemporary threats to forest conservation in Kyereyaaso, Akantanso and Kyekyewere respectively (Tano-Offin forest reserve) (*Source:* participatory mapping workshop, 2014)

The main threat to forest conservation in both forest reserves is illegal chainsaw operations—indicated as stumps on the maps. In the Tano-Offin forest reserve, 78% of the respondents in Akantanso indicated that the major threats to forest reserves are caused by chainsaw operators. Similar high proportions applied to the respondents in Kyereyaaso (64%) and Kyekyewere (56%). Illegal chainsaw operations had been carried out at long distances from the forest boundary. However, Kyereyaaso (Fig. 3, first map) and Akantanso (Fig. 3, second map), have locations without illegal chainsaw operations as the trees are inaccessible due to the hilliness of the area.

Under the protection regime, there are no logging trails because timber harvesting is forbidden. However, a mining company, whose operations would start soon, had constructed a new secondary road linking Kyekyewere (Fig. 3, third map) to the district capital, Nyinahin. Previously, the secondary road leading to Kyekyewere was in a poor condition and it was difficult to transport logs and villagers out of the forest reserve. However, according to villagers, the new road constructed by the mining company could contribute significantly to the proliferation of illegal activities within the forest reserve.

**Fig. 4:**
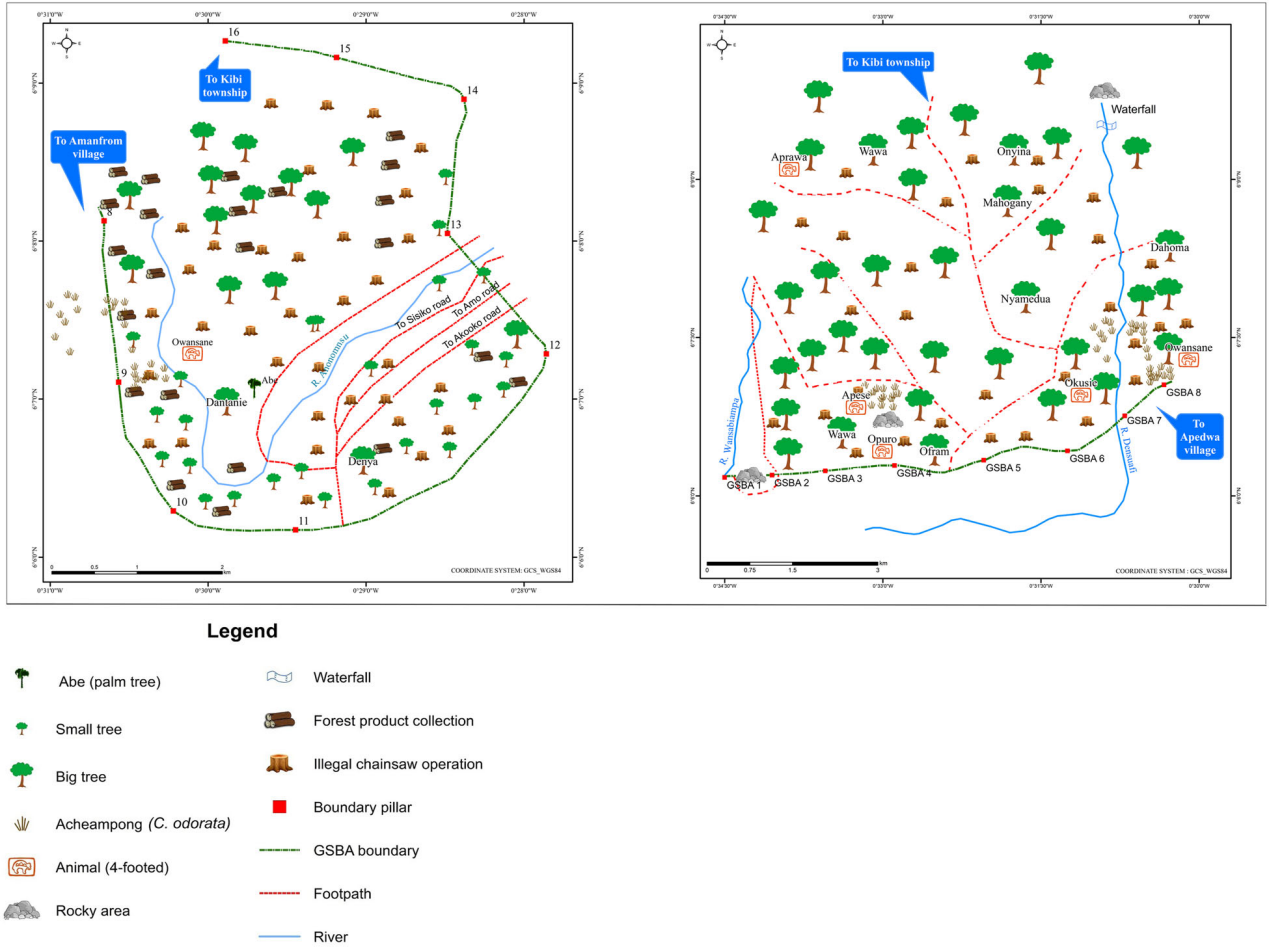
Contemporary threats to forest conservation in Apedwa and Amanfrom respectively (Apedwa forest reserve)^9^ (*Source*: participatory mapping workshop, 2014)

Also in the Apedwa forest reserve, the main group causing threats to forest conservation were chainsaw operators (88% of respondents in Amanfrom and 83% of respondents in Apedwa). Although this reserve has no logging trails as it is under total coverage protection, with the assistance of carrier boys logs can be carried away from the GSBA. Observations revealed that the forest cover in Apedwa village (Fig. 4, first map) was more degraded compared to that of Amanfrom (Fig. 4, second map). The explanation for this is twofold. First, the forest reserve is closer to the village of Apedwa than to Amanfrom.^8^ With longer distances it is more difficult to carry many logs away. Second, the population of Apedwa village is over five times larger than that of Amanfrom (Table 1), which also puts more pressure on the forest.^9^

In both forest reserves, interviewees admitted being aware of the illegal activities of chainsaw operators and attributed the act to unemployment among the youth within the villages. The survey also revealed that in all five villages both natives and settlers are involved in illegal chainsaw operations (indicated by 62% of survey respondents in Akantanso, 70% in Kyereyaaso, 41% in Kyekyewere, 76% in Amanfrom and 84% in Apedwa). Interviewees suggested that villagers go out to seek people with the financial resources to assist in the illegal chainsaw operations. These outsiders then combine forces by partnering with local people who are knowledgeable about where to find the economic tree species. Together, and sometimes assisted by corrupt forestry officials, they are able to conduct illegal chainsaw operations within the forest reserves. Interviewees commented that they are unhappy with the state of degradation in the forest reserves and have reported several times to the forest guard immediately after noticing such illegal activities. For example, in Kyereyaaso, the chief and the District Chief Executive (DCE)^10^ of the area had mounted a barrier at the entrance of the forest reserve to curb the rampant illegal chainsaw operations. However, a community visit in August 2014 revealed that the chainsaw operators had destroyed the barrier.

### Spatializing Minor Threats to Forest Conservation

Minor threats to forest conservation identified in the Tano-Offin forest reserve were illegal farms and wildfires. The forest cover in Kyereyaaso (Fig. 3, first map) is more degraded than in Akantanso (Fig. 3, second map) because of the presence of illegal farms. The explanation is similar as for the Apedwa reserve: the distance covered by a footpath from Akantanso to the forest reserve is longer compared to that of Kyereyaaso, and too far for community members to cover for farming (observation during the transect mapping and reconnaissance survey, February 2014). In addition, Akantanso had a smaller population compared to Kyereyaaso (see Table 1).

The map by the knowledge holders from Kyereyaaso also revealed that the illegal farms are located in places where the forest cover had been destroyed by wildfires. Such locations are also closer to the forest boundary where their farms are located. This observation was the same for Kyekyewere where the illegal farms were closer to the community. However, the illegal farms in Kyekyewere (Fig. 3, third map) are more widespread within the GSBA compared to Kyereyaaso (Fig. 3, first map).^11^ Villagers in Kyekyewere claimed that the FSD had not demarcated the forest boundary with pillars since the reserve was created. However, the population had increased since then, which they considered to be the reason for creating illegal farms. The villagers were also aggrieved because the FSD had refused to grant them portions of the reserve for reforestation schemes^12^, whereas these parts had been destroyed two years ago by wildfires. As a result, Tropenbos Ghana (an NGO) began an initiative in the village to plant trees with artisanal millers. However, this initiative did not yield fruitful results as the FSD refused to grant the village portions of the reserve for reforestation purposes. Being located in a strict protection regime, no timber harvesting rights are permitted and villagers are only allowed into the forest reserve to collect non-timber forest products (NTFPs) for subsistence.

Minor threats to forest conservation identified in the Apedwa forest reserve are the presence of invasive species and the collection of NTFPs. In Amanfrom the knowledge holders identified the presence of ‘acheampong’ (*Chromolaena odorata*) as a threat mainly in places where extensive logging had occurred. In Apedwa, the collection of NTFPs was identified as a threat to forest conservation, as this was done in commercial quantities. A risk of wildfires existed as the forest boundary in both villages is blocked with weeds, as it has been neglected for a long time due to the non-functioning of the CBAGs that are assigned with the task to clear forest boundaries. This observation was the same for Kyekyewere (strict protection regime) in the Tano-Offin reserve, where portions of the GSBA boundary had been blocked by weeds as well. Generally, the two villages had less interest in the forest as they did not receive any social responsibility agreement benefits.^13^

Other groups of people identified as doing harm to the forest include hunters, farmers, timber contractors, corrupt forestry officials and NTFP collectors. Respondents who indicated hunters and farmers as the group causing threats attributed the cause of wildfires within the forest reserves to these actors. During their activities, these groups light fires for purposes such as cooking, land clearing, and in some cases trapping animals (e.g., rats). Afterwards, they often forget to put the fire out, which results in wildfires. In Akantanso and Kyereyaaso (both under a production regime), timber contractors are considered as causing threats to the forest reserves as they over-logged trees within a compartment. Villagers claimed that these activities are carried out under the eyes of corrupt forestry officials who are present to record every logging activity. NTFP collectors are blamed for causing threats to both plant and animal products within the forest reserves because they collect these products in larger quantities for trade instead of subsistence, although the law only permits the latter in protected forest reserves. This practice continues without replacement and has led to the depletion of the forest resources.

### Mapping the Future

**Fig. 5:**
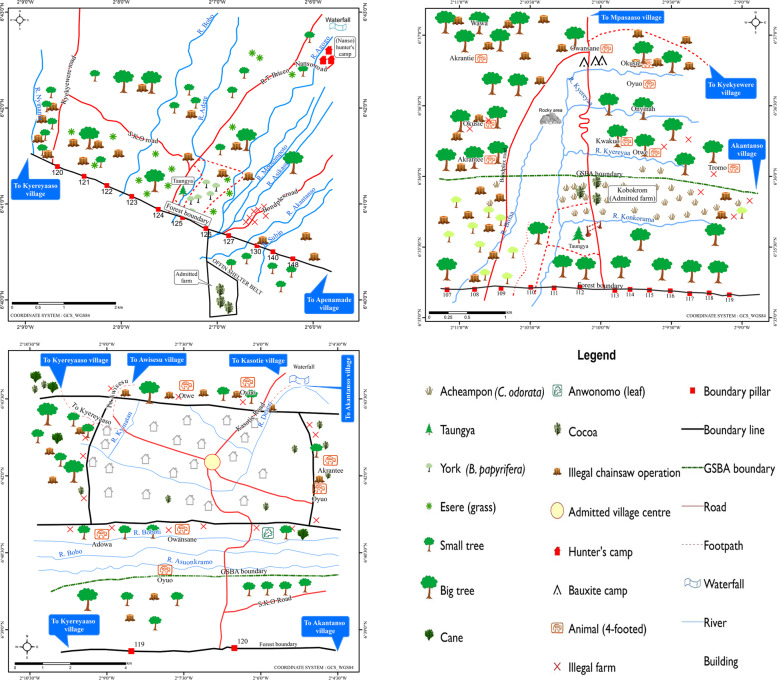
Anticipated forest condition (2024) in Kyekyewere, Akantanso and Kyereyaaso respectively (Tano-Offin forest reserve)^14^ (*Source*: participatory mapping workshop, 2014)

To gain an understanding of the underlying drivers of deforestation as a guide to forest conservation planning, local knowledge holders also mapped the anticipated condition of the forest in 10 years’ time (2024). By 2024, the knowledge holders in Akantanso (production regime) anticipate the invasion of acheampong (*Chromolaena odorata*), esere (grass), and paper mulberry (*Broussonetia papyrifera*), locally known as york, in places destroyed by wildfires (Fig. 5, second map). Due to community expansion with increasing population, the knowledge holders from Akantanso also anticipate the presence of illegal farms in their reserve as there was even not enough land to farm in 2014. Like in Akantanso, in Kyereyaaso (production regime) they anticipate the invasion of acheampong and york in 2024 in places destroyed by wildfires (Fig. 5, third map). They also anticipate more illegal chainsaw operations within the reserve, indicated on the maps by stumps. They also expect greater presence of illegal farms as the community expands. However, they hoped that by 2024 the trees in the new taungya areas will have matured so they can reap their benefits; hence, the presence of more matured trees close to the forest boundary.

In Kyekyewere (strict protection regime), participants anticipated that the village will have expanded significantly into the forest reserve by 2024 (Fig. 5, first map). During the training workshop for knowledge holders in 2014, participants indicated that Kyekyewere currently lacks farmlands for food and cash crops, which threatens the survival of future generations (see also Derkyi et al. 2013). Previously, cocoa could not thrive in the community, but they hope that farming techniques will be available by 2024 which would enable cocoa to survive in the forest reserve.^14^

**Fig. 6:**
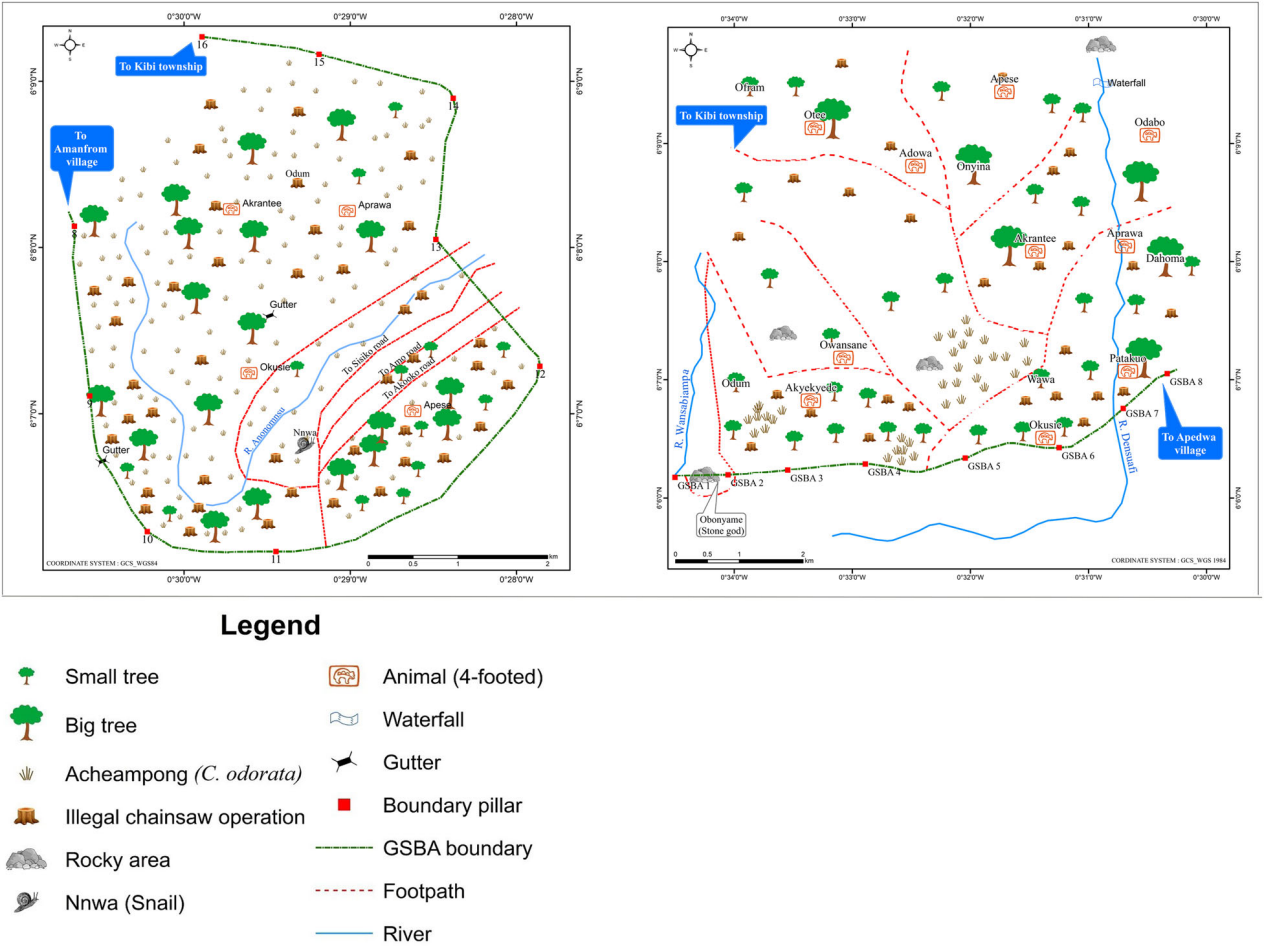
Anticipated forest condition (2024) in Apedwa and Amanfrom respectively (Apedwa forest reserve) (*Source*: participatory mapping workshop, 2014)

In both Apedwa (Fig. 6, first map) and Amanfrom (Fig. 6, second map) near the strictly protected Apedwa forest reserve, the knowledge holders expect significant changes in forest cover by 2024. Those in Apedwa anticipate widespread invasion of acheampong (*Chromolaena odorata*) and increased illegal chainsaw operations, resulting in more stumps and smaller trees in the forest reserve.

## Discussion

### Local Spatialized Knowledge and the Effectiveness of Forest Governance

This paper aimed to make clear how spatializing local knowledge using participatory spatial knowledge tools and PGIS can be used to provide a locally embedded assessment of threats to forest conservation in Ghana. Several other studies document how PGIS enabled communities to map their spatial knowledge for conservation and planning (Fagerholm et al. 2012, 2019; Klain and Chan 2012; Plieninger et al. 2013; McCall 2021). Like our study, these examples highlight how the PGIS approach enabled new undocumented information to be shared among various actors. First, the maps generated by the knowledge holders demonstrated that the participatory spatial knowledge production tools provided a suitable platform for combining local knowledge with geospatial technologies (scientific knowledge). The PGIS approach contributed to the overall knowledge of forests as the knowledge holders not only sketch-mapped the threats to forest conservation, such as the distribution of invasive species and illegal activities, but also provided information on the people perceived to be causing such threats. Further, a comparative assessment of the two forest reserves revealed that minor threats under different conservation status and management regimes vary. For example, illegal cocoa farming and wildfires were identified only in the partially protected forest reserve, suggesting easier access and less enforcement. However, illegal timber operations occurred in both settings, often in collaboration with corrupt FSD officials. Information of such threats and the perpetuators is not available in the records of forest inventories conducted by the FSD within the forest reserves.

Second, mapping the anticipated trends regarding the condition of the forest reserves in 10 years’ time (2024) provided insight into the underlying drivers of deforestation and forest degradation, and can be considered a form of qualitative scenario mapping (c.f. Asubonteng et al. 2021, this issue). The maps of the future suggest that the GSBA concept of preserving tracts of forest for posterity as close to their natural conditions as possible is likely to collapse if the identified threats to forest conservation—illegal chainsaw milling, illegal farming and the spread of invasive species like *Chromolaena odorata* (‘acheampong’)—persist. This proved to be useful as the actors were made aware of the long-term implications of the threats to forest conservation. Actors were made to visualize and perceive the forest condition in the longer term and the need to address the threats to the condition of the forest. The PGIS approach made local spatial knowledge explicit in maps and narratives, highlighting the usefulness of a spatial approach in forest conservation. This suggests that forest governance can become more effective by incorporating local people and their context-embedded spatial knowledge.

Third, the validation workshops held after the mapping exercise enabled spatially explicit discussions among the knowledge holders and the entire communities. These meetings promoted knowledge sharing about the forest resource base among community members and knowledge holders. Through the exchange of knowledge all learned new things by helping each other understand the maps and in some instances correct the information that was represented on them. For example, the location of the threats within the forest reserves was not known by community members who had little or no interaction with the forest reserves. The benefits of the PGIS approach made explicit through visualization and communication helped improve the forest knowledge base of communities as a whole, which illustrates the desirability of organizing such validation meetings.

The question we want to address in the rest of this discussion is whether and under what conditions using participatory geospatial tools and spatializing community knowledge on forest threats can also contribute to more *inclusive* forest governance.

### Spatializing Community Knowledge and Inclusive Forest Governance

Several studies have analyzed how specific environmental problems can be solved by using participatory mapping and geospatial technologies (Pfeffer et al. 2013; Forrester et al. 2015; Robinson et al. 2016; Young and Gilmore 2017; Fagerholm et al. 2019). But how can these participatory processes ensure inclusive decision-making in landscape management and governance more broadly? In general terms, ensuring inclusiveness of participation in knowledge production and environmental governance implies that due consideration is given to the terms of inclusion and exclusion and their implications for representation, citizenship, and democracy (Bäckstrand 2003; Elwood 2006; Turnhout et al. 2010; Anokye 2013).

First, some preconditions are to be met to ensure that the views of an entire community and not only those of a privileged few are represented in knowledge co-production and decision-making. This implies that due consideration is given to the selection of local spatial knowledge holders. This study revealed some possible limitations in this respect. In the Ghanaian context, as elsewhere in the global South, it is important to respect traditional authorities and local customs when entering a community (c.f. Ros-Tonen and Derkyi 2018). Any engagement with local communities, including the selection of local knowledge holders, is to be done through these authorities (chief and elders). There are two risks involved here. The first is that the researcher has no influence on the selection process other than the ability to communicate the selection criteria, which may compromise the representativeness of the participants and confine the selection to those with a higher status in the community—a risk that may also apply to this study. An associated risk is elite capture. Other studies have observed this; both in p-mapping and PGIS (Bauer 2009; Verplanke et al. 2016; Sandström et al. 2020) and in participatory and community-based forestry (Schreckenberg and Luttrell 2009; Vyamana 2009; Chomba et al. 2015). As a result, spatializing local knowledge may (unintendedly) result in elite capture of knowledge, information and ‘professionalization’ processes that give the selected knowledge holders a privileged position in formal forest management (Lund 2015). Several studies have, however, shown that initial elite capture can be overcome through resistance by those who are excluded (e.g., Lund and Saito-Jensen 2013) and/or deliberate (government) measures that ensure equitable representation in local decision-making bodies (e.g., Saito-Jensen et al. 2010). In-depth consultation of community members as part of prior stakeholder analysis may make the selection process more inclusive (McCall and Dunn 2012; Brown and Fagerholm 2015), while validation workshops with the entire community as described above may contribute to knowledge sharing and collectivization of local spatial knowledge.

Second, this study showed the importance of meeting preconditions related to trust, language and location that earlier studies have also indicated (e.g., McCall and Dunn 2012; Pfeffer et al. 2013). Consultations and knowledge co-production processes should be held in a language understood by all (see also Norström et al. 2020; Asubonteng et al. 2021, this issue). Also facilitation by an impartial outsider (researcher or otherwise) may contribute to inclusive engagement of all community members (Balint and Mashinya 2006; Sayer et al. 2016; Reed et al. 2019). It is important to organize consultations and meetings in locations accessible to all community members, including marginalized ones (McCall and Dunn 2012; Pfeffer et al. 2013; Sessin-Dilascio et al. 2015; Ros-Tonen et al. 2018; Reed et al. 2019).

Third, the selection of community actors already involved in local forest management as spatial knowledge holders seems to be an evident choice, as they are most conversant with forest management issues. Moreover, such actors can serve as bridging actors who pass on information and facilitate joint learning and collective action and decision-making among all community members (Green et al. 2015; De Kraker 2017). Evidence from this study showed, however, that such local institutions—in Ghana the community forest committees (CFCs) and CBAGs—may be non-functioning. In that case, other legitimate local bodies should be identified and consulted (see also Turner et al. 2016); in Ghana, for instance, the District Assemblies.^15^

Fourth, an important dimension of inclusion concerns the ownership of local spatialized knowledge. If local communities and their representatives—the Chiefs and elders—can effectively claim ownership of the jointly produced maps, they can use them as boundary objects or ‘negotiation facilitators’ (Alin et al. 2013) in various decision-making processes with other actors in forest and landscape governance (McCall and Minang 2005; Somuah 2018). They can use the maps to negotiate the ways in which potential forest benefits are shared, claim resources, or limit the threats to forest conservation by pointing to areas of illegal activities. If local communities cannot effectively retain the maps as their property, outsiders can use (or misuse) community knowledge to their own advantage, without sharing the benefits with local communities, and bypassing processes of FPIC about resource exploitation. This is an important prerequisite for inclusive knowledge production and forest governance, and implies due consideration of the ethics of participatory mapping and PGIS (McCall 2003; Chambers 2006; Rambaldi et al. 2006; McCall and Dunn 2012; Fagerholm 2014; Tuulentie et al. 2020).

Limitations should however be acknowledged. Effective consultations with local people are time-consuming and budget-intensive, so trade-offs exist as most projects have limited time and financial resources (Pham et al. 2015). Further research could shed light on how such limitations could be overcome and particularly what role knowledge-brokering and boundary organizations could play in this respect (Turnhout et al. 2013; Hering 2016; McGonigle et al. 2020). There is also a need to consider research on how remote sensing analysis and participatory approaches can be combined to enhance the effectivity of both; with participatory approaches ensuring a firm contextual grounding of the findings and inclusive landscape governance, and remote sensing allowing for the validation of local spatial knowledge.

## Conclusion

This paper has shown how participatory spatial knowledge tools and PGIS can mobilize community-embedded spatial knowledge and contribute to an integrated assessment of threats to forest conservation in Ghana’s high forest zone. They provide useful tools for location-specific, spatially aggregated data mapping and discussions among various actors about future strategies. As such, spatializing community knowledge should be recognized in the implementation of forest policies and conservation planning. The discussion showed that several conditions regarding the selection of knowledge holders, language, location and community ownership need to be met to ensure the inclusiveness of participatory spatialized knowledge production and landscape management decision-making. Considering the importance of forests for local livelihoods, forest protection and restoration remain important. This paper has shown how spatialized community-embedded knowledge can help achieve this in a more effective and inclusive manner.

## Supplementary information

Supplementary Material

## Footnotes

1 Ghana’s forest reserves include permanently protected areas (22%), production forest (47%), convalescence areas that are temporarily set apart from production forests to recover their stocks (7%) and conversion areas destined to replanting with timber plantations (8%). The remaining portion is classed as ‘not inventoried’ (16%) (FAO 2010; Ankomah et al. 2019). For the purpose of this study convalescence areas were considered as being part of the production regime.

2 The Ghana Forestry Commission created the community biodiversity advisory groups (CBAGs) and the community fire volunteer squads (CFVSs) as co-management institutions at community level when it demarcated the GSBAs, with a view to getting community support for cleaning the reserve boundaries and wildlife control (Derkyi 2012).

3 A range supervisor together with the forest guard assisted with the reconnaissance survey because of their in-depth knowledge of the area and good contacts with community leaders and community groups involved in collaborative forest management.

4 Unit Committees are the lowest statutory administrative units in Ghana, responsible for the registration of births and deaths, monitoring the implementation of self-help and development projects, acting as focal point to discuss local problems, overseeing the performance of District Assembly members, etc. (Legislative Instrument 1967, Fourth Schedule, Regulation 25 (2010) cited in Botchwey 2017:181).

5 Mental maps or sketch maps are a representation of an individual or group’s cognitive map, are usually hand-drawn from memory on paper or computer-assisted, and do not use georeferencing or a consistent scale (Brennan-Horley 2010; Pánek 2015). Transect maps are as the result of transect walks along a territorial cross-section and describe the location and distribution of landscape features and resources, and the main vegetation types and land uses (Buck and Scherr 2009; Pánek 2015).

6 Scale mapping is used where accurate and scale maps are available; Global Positioning Systems (GPS) mapping is done by using a GPS for accurate mapping of locations within and around a community (Pánek 2015). Scale and GPS mapping are both useful for communicating community information to decision-makers because they use recognized cartographic protocols (IFAD 2009; Pánek 2015).

7 Although the p-maps were drawn in 2014, the findings regarding the perceived threats are still topical, as shown in a recent study by Ankomah et al. (2019).

8 During the transect walks we observed that community members accessed the forest reserves using footpaths (see Figs. 3–6). Following the footpaths revealed varying distances to the forest boundary, with Akantanso (Tano-Offin forest reserve) being located at one mile (1.7 km) from the forest boundary; Kyereyaaso at 0.5 mile (0.85 km); and Kyekyewere at 1–1.5 miles (1.7–2.55 km). The longer distance of the latter is rather surprising for a village in the middle of a GSBA, but can be explained by the farms surrounding the village. In the Apedwa forest reserve, Amanfrom is located at a distance of 4.5 miles (7.65 km) from the GSBA boundary, whereas Apedwa community lies 2 miles (3.4 km) from the boundary.

9 See Supplementary Material, Annex 1 for the meaning of the local plant and animal names on the maps.

10 The District Chief Executive (DCE) is the principal representative of the central government in the district, responsible for the executive and administrative functions of the District Assembly (Republic of Ghana 1992).

11 In Kyekyewere, some villagers indicated that the FSD has a so-called rapid patrol team that destroyed their illegal farms. Once farmers have exhausted their farmlands, they establish such farms in the GSBA in a scattered way so that in case of destruction by the rapid team some will be left for families to survive on.

12 The Ghana Forestry Commission has a reforestation scheme in co-management with villagers—the modified taungya system—in which participants are allowed to interplant food crops as long as the canopy has not yet closed and light is sufficient for food crops to grow. Farmers are entitled to 100% of the food-crop proceeds, and to 40% of the timber revenues in return for their work in tree planting and maintenance. Moreover, 5% of the revenues accrue to the communities for their assistance in wildfire prevention and monitoring illegal use. Villagers are keen to participate in the scheme as it relieves the pressure on scarce farming land (Agyeman et al. 2003; Derkyi 2012; Ros-Tonen et al. 2014; Acheampong et al. 2016; Foli et al. 2018).

13 As stipulated in the 1997 Timber Resources Management Act 547, section 3(3)(e), holders of legal logging permits (so-called Timber Utilization Contracts or TUCs) have to pay a minimum of 5% of stumpage fee in the form of amenities, services or benefits to communities or inhabitants of timber utilization areas (Ros-Tonen and Derkyi 2018).

14 Preliminary results of a trend analysis using remote sensing data/GIS for the study areas, using satellite imagery for 2020, confirm the degradation of the forest cover that the local spatial knowledge holders projected for 2024. Detailed results will be submitted as another paper.

15 To facilitate the decentralization process in Ghana, the Local Government Act (Act 462) designates District Assemblies (DAs) as the main decision-making body of local government (Local Government Act, 1993). The DAs have responsibilities of budgeting, legislating, and planning at the local level (Ibid.). Regarding the forestry sector, the DAs collaborate with the FSD to monitor and control illegal chainsaw activities as well as in the enforcement of byelaws on wildfires.
