# Supplementary material for: Local Spatialized Knowledge of Threats to Forest Conservation in Ghana’s High Forest Zone
Source: Environ Manage. 2021 Mar 22;68(5):738–54. doi: 10.1007/s00267-021-01455-0 (PMC8560677; doi:10.1007/s00267-021-01455-0)
Supplement: Supplementary file 1 — Supplementary Material [file 267_2021_1455_MOESM1_ESM.docx]

**Annex 1** Mapped plant and animal species

| Local name | English/  Scientific name | Main use | Appears on the maps of |
| --- | --- | --- | --- |
| *Tree species*^a^ | | | |
| Dahoma | *Piptadeniastrum africanum* | Timber for exterior use, e.g. sleepers, outdoor furniture, structures | Amamfrom (Fig. 4), Kyeraaso (Fig. 5) |
| Dantanie (Dante) | *Nesogordonia papaverifera* | High quality joinery,  cabinet work, bench  tops, boat components,  decking, turnery | Apedwa (Fig. 4) |
| Denya | *Cylicodiscus*  *gabunensis* | Heavy construction,  marine construction,  dock work, bridges,  ship and boat  building and other heavy duty applications | Apedwa (Fig. 4) |
| Mahogany | *Khaya ivorensis* | High quality joinery, cabinet work, furniture, doors, guitars, etc. | Amamfrom (Fig. 4) |
| Nyamedua | *Alstonia boonei* | Trim interior, veneer, carving and used locally as aphrodisiac, for medicinal purposes (e.g. treating malaria, diabetes)^b^ | Amamfrom (Fig. 4) |
| Odum | *Milicia excelsa* | Exterior and interior joinery, cabinet work, garden furniture, flooring, etc. | Apedwa (Fig. 6), Amamfrom (Fig. 6) |
| Ofram | *Terminalia superba* | Rotary veneer, plywood, blockboard, interior joinery, panelling, moulding and trim, selected furniture | Amamfrom (Fig. 4, 6), |
| Onyina | *Ceiba pentandra* | Used for its kapok, in stuffing matresses, etc.^c^ | Amamfrom (Fig. 4), Kyeraaso (Fig. 5) |
| Wawa  Otee | *Triplochiton scleroxylon*  *Pycnanthus angolensis* | Rotary veneer, plywood,  blockboard, interior  joinery, panelling,  moulding  Timber species used for construction | Amamfrom (Fig. 4, 6), Kyeraaso (Fig. 5)  Amanfrom (Fig. 6) |
| *Animal species*^d^ | | | |
| Akyekyede | Tortoise  (*Kinixys erosa*) | Food (soup) | Amamfrom (Fig. 4, 6) |
| Adowa | Royal antelope (*Neotragus pygmaeus*) | Food (soup) | Amamfrom (Fig. 4) |
| Ahweaa | Cusimanse  (*Crossarchus obscurus*) | Food (soup) | Amamfrom (Fig. 4, 6) |
| Akrantee | Grasscutter (*Thryonomys swinderianus*) | Food (soup) | Amamfrom (Fig. 4, 6), Kyeraaso (Fig. 5), Apedwa (Fig. 6) |
| Apese | African brush-tailed porcupine (*Atherurus africanus*) | Food (soup) | Amamfrom (Fig. 4, 6), Apedwa (Fig. 6) |
| Aposo | Bosman's Potto (*Perodicticus potto*) | Food (soup) | Amamfrom (Fig. 4) |
| Aprawa | Ground pangolin (*Manis temminckii*) | Food (soup) | Apedwa (Fig. 6), Amamfrom (Fig. 6) |
| Kwakuo | Lowe’s Monkey  (*Cercopithecus campbelli lowei*) | Food (soup) | Amamfrom (Fig. 4), Kyeraaso (Fig. 5) |
| Nnwa | Snails (*Gastropod*) | Food (soup) | Apedwa (Fig. 6) |
| Odabo | Bay duiker  (*Cephalophus dosalis*) | Food | Amamfrom (Fig. 4) |
| Okusie | Rat (*Cricetomys gambianus*) | Food (soup) | Kyeraaso (Fig. 5), Apedwa (Fig. 6), Amamfrom (Fig. 6) |
| Opuro | Squirrel (several species) | Food (soup) | Amamfrom (Fig. 4) |
| Owea | Western tree hyrax (*Dendrohyrax dorsalis*) | Food (soup) | Amamfrom (Fig. 4) |
| Owansane | Bushback (*Tragelophus scriptus scriptus*) | Food (soup) | Amamfrom (Fig. 4, 12) |
| Otwe | Maxwell's duiker (*Philantomba maxwellii*) | Food (soup) | Amamfrom (Fig. 4, 12) Kyeraaso (Fig. 5) |
| Oyuo | Black duiker (*Cephalophus niger*) | Food (soup) | Amamfrom (Fig. 4), Kyeraaso (Fig. 5) |
| Patakuo | Hyena  (*Crocuta crocuta*) | Food (soup) | Amamfrom (Fig. 4) |

^a^ Based on Ghana Forestry Commission n.d. and Triton n.d.. ^b^ Based on Adotey et al. 2012. ^c^ Based on <https://plants.jstor.org/compilation/Ceiba.pentandra>. ^d^ Based on Somuah 2018.

**References:**

Ghana Forestry Commission Helping the timber industry to come to the right decision. <https://ghanatimber.org/applications.php?page=13&sort=&search=&order=asc> . Accessed 3 Nov 2020

Triton Ghana hardwoods.

<http://tritontimber.com/wp-content/uploads/2012/08/WoodDatabaseGHANAcompletelist.pdf> Accessed 3 Nov 2020
